# Case–control study of HLA-G promoter methylation status, HPV infection and cervical neoplasia in Curitiba, Brazil: a pilot analysis

**DOI:** 10.1186/1471-2407-12-618

**Published:** 2012-12-24

**Authors:** Anna Gillio-Tos, Maria da Graça Bicalho, Valentina Fiano, Chiara Grasso, Valentina Tarallo, Laura De Marco, Morena Trevisan, MarinaBarbaradeSousa Xavier, Renata Slowik, Newton S Carvalho, Carlos A Maestri, Hadriano M Lacerda, Daniela Zugna, Lorenzo Richiardi, Franco Merletti

**Affiliations:** 1 Department of Medical Sciences, Unit of Cancer Epidemiology – C.E.R.M.S, University of Turin, Turin, Italy; 2 Laboratory of Immunogenetics and Hystocompatibility, Federal University of Paranà, Curitiba, Brazil; 3 Department of Gynecology and Obstetrics, Federal University of Paraná, Infectious Diseases in Gynecology and Obstetrics Sector, Hospital de Clínicas, Curitiba, Brazil; 4 Department of Cervical Pathology, Hospital Erasto Gaertner, Curitiba, Brazil; 5 Centre for Oncologic Prevention, Turin, Italy

**Keywords:** HPV, Cervical cancer, HLA-G, Methylation

## Abstract

**Background:**

The causal association between persistent human papillomavirus (HPV) infection and cervical cancer has been established, but the mechanisms that favor HPV persistence in cervical cells are still unknown. The diminished capability of the immune system to control and resolve HPV infection is one of several hypotheses. The tolerogenic protein HLA-G has shown aberrant expression in a variety of cancers, which has been suggested as a mechanism for tumor escape from immunosurveillance. In the present study we evaluate the role of epigenetic modification (promoter de-methylation) of the HLA-G gene on susceptibility to HPV infection and development of high-grade cervical lesions.

**Methods:**

A case–control study was carried out in Curitiba, Brazil, between February and June 2010. A total of 789 women aged 15–47 years were recruited: 510 controls with normal cervical cytology, and 279 cases with histologically confirmed cervical intraepithelial neoplasia grade 2 (CIN2, N = 150) or grade 3 (CIN3, N = 129). All women were administered a questionnaire by interview, which collected information on demographic and lifestyle factors, and a cervical sample was collected. HPV DNA detection was performed by GP5+/GP6+ primer-mediated PCR. HPV-positive samples were genotyped by multiplex PCR. A pilot analysis of HLA-G promoter methylation was carried out in a subset of the study population (96 cases and 76 controls) by pyrosequencing. HLA-G methylation and HPV infection status of cases and controls were compared, and confounding factors were computed by *t* Student and non-parametric Wilcoxon tests. Comparison of HLA-G methylation between cases and controls was assessed by the Bonferroni correction. The association of HLA-G methylation with CIN2/3 was evaluated by logistic regression.

**Results:**

HPV prevalence was 19.6% in controls and 94.3% in CIN2/3 cases. HPV16, 31, 33, 35 and 18 were the most prevalent types. Methylation analysis of seven CpGs in the HLA-G promoter did not reveal any spontaneous de-methylation events in CIN2/3 cases (mean proportion of methylation: 75.8%) with respect to controls (mean 73.7%; odds ratio 1.01, 95% confidence interval 0.96, 1.07).

**Conclusions:**

This study did not support the hypothesis that spontaneous de-methylation events in the HLA-G promoter play a primary role in promoting escape from immunosurveillance in the development of precancerous cervical lesions.

## Background

Cervical cancer is the third most common cancer and the fourth cause of cancer mortality in women worldwide [1]. Screening programs in industrialized countries have drastically reduced the incidence and mortality of cervical cancer, and research in the last decades has greatly improved our capability to detect the etiologic viral component of the disease, human papillomavirus (HPV), and contributed to a decrease in rates of progression to cervical cancer.

Nevertheless, cervical cancer remains a public health issue. Several unresolved aspects of the natural history of the disease also remain. Among these is the mechanism behind the development of cervical cancer. Indeed, only a small proportion of women infected with HPV ever develop cervical cancer; in most women the infection regresses spontaneously. While the causal association between persistence of HPV infection and risk of developing cervical lesions is recognized [2], the events that promote or prevent this persistence in cervical cells has not yet been identified. Several hypotheses exist, one of which is the capability of the immune system to control and resolve HPV infection. One recently considered hypothesis focuses on human leucocyte antigen-G (HLA-G), a tolerogenic protein involved in the control of immune response, due to its reported aberrant expression in a wide variety of cancer cells [3-6]. HLA-G is a non-classical gene of the major hystocompatibility complex that codes for a protein involved in immunosuppressive mechanisms. HLA-G inhibits cell-mediated immunity through interaction with receptors expressed on lymphoid, myeloid and natural killer cells [7,8]. It plays a primary role in immune tolerance, and has been widely described in fetal-maternal tolerance [9]. The HLA-G protein is physiologically present in fetal [10] and immature (thymus [11]) cells, and in a small number of adult tissues [5,11-13], but it is not commonly expressed in mature normal cells. HLA-G re-expression has been suggested as a mechanism of viral [14] and tumor [3-6] escape from immunosurveillance. Recent evidence [15] showed that HLA-G expression increased with grade of precancerous cervical lesions, with the highest expression found in cervical cancer. Since promoter methylation has been described as one of the crucial mechanisms that regulate gene expression [16], occurrence of spontaneous de-methylation events in the HLA-G promoter was postulated to explain the re-expression of this protein in adult cells. De-methylation events have been reported in the HLA-G promoter of ovarian tumor cells compared to normal ovarian epithelial cells [17].

The present study aimed to investigate the association of HPV infection and development of cervical intraepithelial neoplasia grades 2 (CIN2) and 3 (CIN3) with HLA-G promoter methylation in a Brazilian population. Association with the characteristics of the study population was also evaluated.

## Materials and Methods

### Study population and sample collection

A case–control study was set up in the framework of the collaboration among the Unit of Cancer Epidemiology in Turin, Italy; the Laboratory of Immunogenetics and Hystocompatibility (LIGH) in Curitiba, Brazil; the Department of Gynecology and Obstetrics, at the Federal University of Paraná, Infectious Diseases in Gynecology and Obstetrics Sector; and the Department of Cervical Pathology, Hospital Erasto Gaertner, in Curitiba, Brazil. The study was approved by the Ethical Committee for Clinical Research of the Hospital Erasto Gaertner (protocol CEP: 81520–060, P.P No 1943). All participating women were informed about the study purpose and signed an informed consent form.

Women were recruited in Curitiba, where the prevalence of HPV infection and cervical lesions has been reported to be higher than in Turin [18-20]. Under the supervision of the LIGH, women aged 15 to 47 years were recruited: local gynecologists working at three reference centers for cervical cancer screening collaborated to enroll women. Some women were also recruited through awareness campaigns for adhesion to cervical screening. Women over 47 years of age were not included to avoid atrophy or dysplasia associated with menopause, although the suggested impact of menopausal hormonal status on cervical dysplasia due to a weakened immune response, specifically in HPV-positive menopausal women, has not been properly documented.

A total of 789 women were recruited: 510 had normal cervical cytology and were classified as controls; 279 had histologically confirmed CIN2 (N = 150) or CIN3 (N = 129) after loop electrosurgical excision procedure or cold knife conization, and were classified as CIN2/3 cases. A cervical sample was collected from all study women. Cervical cell samples were collected from controls at collection for cytology using the cytobrush provided in the collection kit (Digene sample collection kit, Qiagen, Hilden, Germany), which was then placed into a tube containing sample transport medium (STM, Qiagen). A cervical sample was analogously collected in STM from CIN2/3 cases at loop electrosurgical excision procedure or cold-knife conization.

Study women were administered a questionnaire by interview, which collected information on demographic, sexual and lifestyle factors, including age, education level, ethnic group, age at first sexual intercourse, lifetime number of sexual partners, number of full-term pregnancies, smoking status and number of cigarettes smoked per day. Study women were classified into four ethnic groups based on their replies in the questionnaire: Euro-Descendent, Afro-Descendent, Brazilian Mixed and Asian. There is a general consensus as to the definition of a person of Brazilian Mixed ethnicity in the Genetic Department of the Federal University of Paranà in Curitiba, and following that consensus, all study women with a multiracial origin, i.e., a miscegenation of Euro-Descendent (mostly), Afro-Descendent, Amerindian and East Asian, were classified as Brazilian Mixed [21]. Women were assigned to the corresponding ethnic group following interview at recruitment.

### DNA extraction

Genomic DNA was extracted from cervical cell samples using the commercial purification system QIAmp DNA Mini Kit (Qiagen) according to the manufacturer’s instructions. The final elution in 100 μl of the provided elution buffer was repeated twice to increase the DNA yield. The DNA concentration was evaluated by a Nanodrop spectrophotometer (Thermo Scientific, Wilmington, DE, USA). DNA adequacy was checked by amplification of a β-globin housekeeping gene sequence of 268bp, as previously described [22]. After gel electrophoresis onto a 2% agarose gel stained with ethidium bromide, amplicons were visualized by ultraviolet trans-illumination. The amplicon of the β-globin gene fragment was detected in all the study samples. Purified DNA was stored at −80°C.

### HPV detection

HPV detection was performed in Turin, Italy, on the genomic DNA extracted from cervical samples by consensus primer GP5+/6 + −mediated PCR [23], which allows to detect a broad variety of HPV types. PCR reaction was performed in a total volume of 25 μl containing buffer (KCl) 50 mM, Tris-HC1 10 mM pH 8.3, dNTP 200 μM, MgCl_2_ 3.5 mM, Taq polymerase 1U, GP5+/6+ 50 pmol and DNA 5 μl. The following amplification profile was used: 94°C for 9 min, followed by 40 cycles of denaturation at 94°C for 20s, annealing at 38°C for 30s, extension at 71°C for 80 s. A final extension of 4 min at 71°C was performed.

### HPV genotyping

HPV-positive samples were genotyped by multiplex PCR, in order to detect the seven oncogenic HPV types that are prevalent, and more associated with cervical cancer in Brazil [24]: HPV16, 18, 31, 33, 35, 45 and 52. Multiplex PCR was performed as previously described [25], with the exception of HPV16, for which a different primer set was used [26]. Briefly, the PCR mix was carried out in a final volume of 25 μl containing buffer (KCl) 1X, MgCl_2_ 2 mM, dNTPs 200 μM, 0.4 μM both primers, Taq polymerase 2.5U, and DNA 3 μl. The amplification profile was as follows: 94°C for 4 min, followed by 35 cycles of denaturation at 94°C for 30s, annealing at 56°C for 30 s, extension at 72°C for 45 s. A final extension at 72°C for 4 min was performed.

The set of primers used for the multiplex PCR were as follows: HPV16 sense 5^′^AAGGGCGTAACCGAAATCGGT3^′^, antisense 5^′^CATATACCTCACGTCGCAG3^′^; HPV18 sense 5^′^CACTTCACTGCAAGACATAGA3^′^, antisense 5^′^GTTGTGAAATCGTCGTTTTTCA3^′^;

HPV31 sense 5^′^GAAATTGCATGAACTAAGCTCG3^′^,antisense 5^′^ACATATACCTTTGTTT-GTCAA3^′^; HPV33 sense 5^′^ACTATACACAACATTGAACTA3^′^, antisense 5^′^GTTTTTACACG-TCACAGTGCA3^′^; HPV35 sense 5^′^CAACGAGGTAGAAAGC-ATC3^′^, antisense 5^′^CCGA-CCTG TCCACCGTCCACCG3^′^; HPV45 sense 5GATGGAAAAGTGCATTACAGG3, antisense 5^′^ACCTCTGTGCGTTCCAATGT3^′^;HPV52 sense 5^′^TAAGGCTG CAGTGTGTGCAG3^′^, antisense 5^′^CTAAT AGTTATTTCACTTAATGGT3^′^.

Samples positive at HPV detection, but negative at PCR genotyping were re-tested by reverse-line blot hybridization using the Digene HPV Genotyping RH (Qiagen) commercial kit, according to the manufacturer’s protocol. The kit employed biotynilated primers (GP5+/GP6+) and the assay targets 18 HPV types [27], including those classified by the International Agency for Research on Cancer (IARC) as carcinogenic (Group 1 carcinogen, HPV16, 18, 31, 33, 35, 39, 45, 51, 52, 56, 58, 59), probably carcinogenic (Group 2A carcinogen, HPV68), and as possibly carcinogenic to humans (Group 2B carcinogen, HPV26, 53, 66, 73, 82) [28]. PCR biotinylated products (10 μl) were denatured and hybridized with type-specific oligonucleotide probes immobilized as parallel lines on nitrocellulose membrane strips. The hybrids were detected with alkaline phosphatase–streptavidin conjugate and substrate (5-bromo-4-chloro-3-indolylphosphate and nitroblue tetrazolium), resulting in a purple precipitate at positive probe lines. After drying, the strips were analyzed by visually comparing them with the interpretation grid supplied in the kit; the presence of a clearly visible line was considered a positive reaction.

A biotinylated poly (dT) control for conjugate reaction was applied to each strip to ensure the validity of the test and proper alignment of the strips on the interpretation sheet.

### HLA-G methylation – pilot analysis

The analysis of the HLA-G methylation was performed as a pilot analysis on the first set of samples shipped to Italy from Brazil (N = 172, 76 controls and 96 CIN2/3 cases). The analysis was performed through bisulfite modification and pyrosequencing.

#### Bisulfite modification

Sodium bisulfite modification converts unmethylated cytosine into uracyl, but leaves the methylated cytosines unchanged. Genomic DNA (1μg) from cervical samples was converted using the EpiTect Bisulfite commercial kit (Qiagen) according to the manufacturer’s instructions.

#### Pyrosequencing

Pyrosequencing was used to evaluate HLA-G methylation status and to quantify the methylation of each individual CpG investigated. It was performed on a Pyromark Q24 using PyroMark Gold Q24 (Qiagen) reagents. The assay allowed the quantification of methylation of the seven CpGs we sited in the HLA-G promoter, using primers designed with the Pyromark Assay Design software (Qiagen) according to the HLA-G reference sequence GenBank J03027.1. Preliminary PCR was performed, targeting a 441 bp sequence of the HLA-G promoter, using primers with the following sequences: sense 5^′^GGGAGGTAGGGAGTTTAGTT-TA3^′^, antisense Biotin-5^′^CCATAACCACCATCCTTAAC3^′^. The primer antisense is biotinylated to allow binding to sepharose beads during the subsequent pyrosequencing process. To improve efficiency, three different pairs of sequencing primers that targeted 2, 3 and 2 CpGs, respectively, were employed: Sequencing primer 1 (2 CpGs, positions 350 and 428) 5^′^GGAGTTTAGTTTAGGGATAG3^′^, Sequencing primer 2 (3 CpGs, positions 494, 512 and 523) 5^′^ATTTAGGGAGATATTGAGA3^′^, Sequencing primer 3 (2 CpGs, positions 573 and 598) 5^′^GGGTTTTAGGTTTTATAGG3^′^.

The preliminary PCR reaction was performed in a total volume of 35 μl containing buffer (KCl) 1X, MgCl_2_ 2 mM, dNTPs 200 μM, 0.5 μM each primer (antisense biotinylated), Taq polymerase 1.75U and 6 μl converted DNA with the following cycling profile: 95°C for 1 min followed by 45 denaturation cycles at 54°C for 1 min, annealing at 56°C for 1 min, extension at 72°C for 1 min, final extension at 72°C for 10 min. Amplicons were analyzed by gel electrophoresis on a 2% agarose gel stained with ethidium bromide and visualized by ultraviolet trans-illumination. The residual PCR product (28 μl) was added to 12 μl of dH_2_O and incubated under shaking with 37 μl of binding buffer pH 7.6 (10 mM Tris–HCl; 2 M NaCl; 1 mM EDTA; 0.1% Tween 20) and 3 ml sepharose beads covered with streptavidin. PCR products were washed with ethanol 70%, denatured with NaOH 0.2 M and re-washed with Tris-Acetate 10 mM pH 7.6. Pyrosequencing reaction was performed in 45 μl of annealing buffer [44.82 μl of 20 mM Tris-Acetate + 5 mM MgAc_2_ and 0.18 μl of sequencing primer (0.3 μM)].

Quantitative methylation results were expressed as the mean of the methylation percentage of all seven CpGs investigated.

The individual methylation percentage for two CpGs, one located in a binding site for the transcription factor specificity protein 1 (Sp1), and the other located in the *enhancer* region, were also evaluated for their association with HPV infection and CIN2/3.

### Statistical analyses

Analyses investigating the association between HLA-G methylation, HPV status, and demographic and lifestyle factors were restricted to controls, due to the fact that almost all CIN2/3 cases were HPV-positive. For HLA-G methylation analyses with sufficiently high frequency, the *t* Student test was used, or alternatively the non-parametric Wilcoxon test. To evaluate whether there was a difference in the percentage of methylation between CIN2/3 cases and controls, and to further compare subgroups of the study population (i.e., controls vs. CIN2 cases, controls vs. CIN3 cases, CIN2 cases vs. CIN3 cases), the Bonferroni correction for multiple comparisons was used. Logistic regression models, adjusted for age, education level, ethnic group and smoking status, were fitted to evaluate the effect of HLA-G methylation on the development of cervical cancer [crude and adjusted odds ratios (ORs) are reported].

## Results

### Characteristics of the study population

During the study period 789 women were recruited, including 510 controls and 279 CIN2/3 cases (150 CIN2, 129 CIN3). CIN2/3 cases and controls had a comparable mean age (32 years for both), education level, age at first sexual intercourse (16 years for cases, 17 years for controls) and number of full-term pregnancies (Table 1). The ethnic group Brazilian Mixed showed the highest prevalence of HPV infection in both CIN2/3 cases and controls. Slight differences emerged in the distribution of CIN2/3 cases and controls by ethnic group, sexual factors, and smoking status. The most represented ethnic group in CIN2/3 cases was Brazilian Mixed (58.1%), while in controls it was Euro-Descendent (46.9%). Among CIN2/3 cases, 74.6% reported a total number of partners between 2 and 10, while the corresponding percentage among controls was 53.9%. Current smokers were more common among CIN2/3 cases (34.4%) than controls (15.9%) (Table 1).

**Table 1:** Characteristics of the study population

	**Cases**		**Controls**	
	**N**	**%**	**N**	**%**
**Number**	**279**		**510**	*100 %*
*Controls (normal cytology)*	-		510	
*CIN2*	150	*53.7*	-	
*CIN3*	129	*46.3*	-	
**Mean age**	32 years		32 years	
*Range*	15-47		15-47	
**Education level**				
*Elementary school*	164	*58.8*	262	*51.4*
*Middle school*	93	*33.3*	159	*31.2*
*High school*	8	*2.9*	18	*3.5*
*Missing*	14	*5.0*	71	*13.9*
**Ethnic group**				
*Euro-Descendent*	90	*32.3*	239	*46.9*
*Afro-Descendent*	7	*2.5*	24	*4.7*
Brazilian Mixed*	162	*58.1*	180	*35.3*
*Asian*	1	*0.4*	1	*0.2*
*Missing*	19	*6.8*	66	*12.9*
**Age at first sexual intercourse**		*16 years*		*17 years*
**Lifetime number of sexual partners**				
*1*	42	*15.0*	156	*30.6*
*2-10*	208	*74.6*	275	*53.9*
*>10*	27	*9.7*	79	*15.5*
*Missing*	2	*0.7*	0	*0*
**Number of full-term pregnancies**	*2*		*2*	
*Range*	*0-18*		*0-27*	
**Smoking status**				
*Never smoker*	130	*46.6*	277	*54.3*
*Former smoker*	51	*18.3*	148	*29.0*
*Number of cigarettes/day (mean)*	*7*		*8*	
*Current smoker*	96	*34.4*	81	*15.9*
*Number of cigarettes/day (mean)*	*7*		*8*	
*Missing*	2	*0.7*	4	*0.8*

*Brazilian Mixed: Mixed ethnicity with Euro-Descendent (mostly), Afro-Descendent, Amerindian and East Asian.

### HPV detection and type distribution

We found a HPV prevalence of 19.6% in controls and 94.3% in CIN2/3 cases (Table 2). HPV-positive CIN2/3 cases and controls showed a higher frequency of single HPV infections (cases 66.2%, controls 65%) than multiple infections (cases 32.7%, controls 29%). A proportion of samples could not be typed (cases 1.14%, controls 6%) by our methods. Among the most frequent genotypes (HPV16, 18, 31, 33, 35, 45 and 52, IARC Group 1 carcinogen [28]) a higher prevalence was observed for HPV16, 31 and 33 for both CIN2/3 cases and controls, followed by HPV35, 52, 18 and 45 in CIN2/3 cases, and HPV18, 35, 52 and 45 in controls (Table 3). A cumulative frequency of 9.3% and 4.9% was detected in CIN2/34 cases and controls respectively, for HPV types with a lower prevalence: HPV39, 51, 56, 58 and 59, IARC Group 1 carcinogen; HPV68, IARC Group 2A carcinogen; and HPV26, 53, 66, 73 and 82, IARC Group 2B carcinogen [28].

**Table 2:** Frequency of human papillomavirus (HPV) infection in the study population

	**Cases**		**Controls**	
	**N = 279**		**N = 510**	
	**N**	**%**	**N**	**%**
**HPV-**	16	5*.7*	410	*80.4*
**HPV+**	263	*94.3*	100	*19.6*
***Single infections***	174	*66.2*	65	*65.0*
***Multiple infections***	86	*32.7*	29	*29.0*
***Not typed by our methods***	3	*1.14*	6	*6.0*

**Table 3:** Distribution of selected human papillomavirus (HPV) types in the study population

**High-risk HPV types**^**§**^	**Cases**		**Controls**	
	**N = 279**		**N = 510**	
	**N**	**%**	**N**	**%**
***HPV16***	171 *61.3*	*61.3*	38	*7.4*
***HPV18***	21 *7.5*	*7.5*	9	*1.8*
***HPV31***	50 *17.9*	*17.9*	16	*3.18*
***HPV33***	31 *11.1*	*11.1*	12	*2.38*
***HPV35***	27 *9.7*	*9.7*	8	*1.6*
***HPV45***	13 *4.7*	*4.7*	6	*1.2*
***HPV52***	24 *8.6*	*8.6*	7	*1.4*
Less frequent HPV types*	26	*9.3*	25	*4.9*

^§^ HPV types present in single or multiple infections.

* Includes IARC Group 1 carcinogen types HPV39, 51, 56, 58, 59; Group 2A carcinogen type HPV 68; and Group 2B carcinogen types HPV26, 53, 59, 66, 73, 82.

### Frequency of HPV infection by selected characteristics in controls

Table 4 reports the frequency of HPV infection by selected characteristics among controls. HPV infection was inversely associated with age (p < 0.001), and was positively associated with lifetime number of sexual partners (p = 0.001), smoking status (p = 0.09) and Brazilian Mixed ethnic group (p = 0.09). There was no evidence of association with education level (p = 0.31).

**Table 4:** Frequency of HPV infection by characteristics of controls

	**CONTROLS**	
	**N = 510**	
	**HPV+/N**	**%**
**Age (years)**		
*<25*	36/101	*35.64*
*25-34*	40/201	*19.90*
*35-44*	22/187	*11.76*
*≥45 < 25*	2/21	*9.52*
**Education level**		
*Elementary school*	57/261	*21.84*
*Middle school*	27/159	*16.98*
*High school*	2/18	*11.11*
**Ethnic group**		
*Euro-Descendent*	39/239	*16.32*
*Afro-Descendent*	4/24	*16.67*
Brazilian Mixed*	47/180	*26.11*
*Asian*	0/1	*0*
**Lifetime number of sexual partners**		
*1*	16/156	*10.26*
*2-10*	62/275	*22.55*
*>10*	22/79	*27.85*
**Smoking status**		
*Never smoker*	45/277	*16.25*
*Former smoker*	36/148	*24.32*
*Current smoker*	19/81	*23.46*

*Brazilian Mixed: Mixed ethnicity with Euro-Descendent, Afro-Descendent, Amerindian and East Asian.

### HLA-G methylation status – pilot analysis

We calculated the mean percentage of methylation of the seven evaluated CpGs of the HLA-G promoter, among the 76 controls and 96 CIN2/3 cases included in the pilot analysis. We did not find lower methylation levels among CIN2/3 cases as expected, instead they were slightly higher (Table 5). Demographic and lifestyle factors, specifically smoking status and ethnicity, were not associated with mean HLA-G methylation (data not shown). Moreover, no decrease was found in overall methylation when HPV-positive and HPV-negative controls were compared (data not shown).

**Table 5:** Overall mean methylation percentage in the study population

	**Cases**	**Controls**
	**N = 96**	**N = 76**
	**(% Methylation)**	**(% Methylation)**
7 CpGs* in HLA-G promoter		
***mean methylation***	*75.8*	*73.7*
***Range***	*70 - 83*	*71 - 83*

* CpG sites: position 350, 428, 494, 512, 523, 573, 598 (GenBank: J03027.1).

The CpGs located in regulatory sites of the HLA-G promoter, i.e., the binding site for the transcription factor Sp1 (sequence position 573), and the *enhancer* region (sequence position 598), were also evaluated individually with the aim of highlighting any relevant decrease in methylation that could directly affect gene transcription. The results, stratified for HPV positivity in controls, and for CIN2 and CIN3 in cases, are shown in Table 6. There was no evidence of any differences in the mean methylation percentage between CIN2/3 cases and controls for the CpG located in the binding site for transcription factor Sp1 (crude OR = 1.01, 95% confidence interval [CI]: 0.97,1.06; adjusted OR = 1.01, 95% CI: 0.96,1.07), while the mean methylation percentage for the CpG located in the *enhancer* region was slightly higher in CIN2/3 cases than in controls (crude OR = 1.04, 95% CI: 1.00,1.08; adjusted OR = 1.03, 95% CI: 0.99,1.08). These results were confirmed when the analysis was restricted to HPV-positive controls for both individually analyzed CpGs (binding site for transcription factor Sp1: crude OR = 1.01, 95% CI: 0.96,1.07; adjusted OR = 0.99, 95% CI: 0.93,1.07; *enhancer* region: crude OR = 1.03, 95% CI: 0.98,1.08; adjusted OR = 1.01, 95% CI: 0.95,1.06).

**Table 6:** Mean methylation percentage in specific CpG sites

	**Cases**		**Controls**	
	**N = 96**		**N = 76**	
	**CIN2 HPV+**	**CIN3 HPV + N = 65**	**HPV +**	**HPV-**
	**N = 31**	**(% Methylation)**	**N = 42**	**N = 34**
	**(% Methylation)**		**(% Methylation)**	**(% Methylation)**
CpG (Sp1)* ***mean methylation***	*83.5*	*83*	*82.7*	*82.4*
***Range***	*65-93*	*70-93*	*70-91*	*65 -93*
CpG (enhancer)** ***mean methylation***	*33.8*	*36.3*	*33.8*	*32.5*
***Range***	*21-53*	*19-69*	*21-50*	*20-56*

* CpG site position 573; **CpG site position 598 (GenBank: J03027.1).

When a threshold of the median percentage of methylation (33%) was applied for the CpG located in the *enhancer* region (Table 7), we obtained a higher proportion of CIN2/3 cases (61.5%) than controls (47.4%) with methylation levels over the threshold (crude OR = 1.77, 95% CI: 0.96,3.26; adjusted OR = 1.40, 95% CI: 0.71,2.76).

**Table 7:** **Distribution of the study population according to methylation level of the CpG located in the *enhancer***

CpG (enhancer)*	**Cases**		**Controls**	
	**N = 96**		**N = 76**	
	**N**	**%**	**N**	**%**
***Mean methylation <33 %***	*37*	*38.5*	*40*	*52.6*
***Mean methylation >33 %***	*59*	*61.5*	*36*	*47.4*

*CpG site position 598 (GenBank: J03027.1).

## Discussion

A case–control study was set up in a Brazilian female population to investigate the relationships between HPV infection, prevalence of HPV types, methylation status in the gene promoter of the tolerogenic HLA-G protein and high-grade cervical lesions.

We explored the occurrence of spontaneous de-methylation in the HLA-G promoter as a surrogate of re-expression of the HLA-G protein in HPV-infected cells, as the HLA-G protein is a recognized inducer of a tolerogenic effect and tumor escape from immunosurveillance. By exploring this in a case–control study, our goal was to try and highlight any association of decreased methylation with the carcinogenic process. Indeed, according to the hypothesis of the association with, and role of de-methylation of the HLA-G protein on oncogenic progression, our controls were expected to show high HLA-G methylation, and our CIN2/3 cases were expected to show low HLA-G methylation. We did not include CIN1 in our study, as it is less informative given its high rate of spontaneous regression [29-33]. Similarly, a recent publication exploring the association between HLA-G expression and cervical cancer progression also focused on high-grade lesions only [34].

We did not consider invasive cervical cancer in the present study, since the occurrence of HLA-G expression in cervical cancer cells is still controversial in the scientific literature. Some authors reported variable HLA-G expression in cervical cancer cells [15,35,36], others very low or no expression [37,38], suggesting that if methylation status plays a role in promoting carcinogenesis, it probably acts in the early phases, rather than in the advanced phases of the process. For these reasons we focused our investigation on comparing normal cervical cells with high-grade cervical lesions, in which the carcinogenic process, if it has started, is more frequently active.

The HLA-G gene is silenced under physiological conditions independently from proliferative or differentiative status of normal cells [10,11,39,40]. Therefore the collection of cervical cells by cytobrush should not have biased the results of our methylation analyses even if many dead epithelial cells were present. As expected, we did find high HLA-G methylation levels in normal cervical cells, which in this context can be considered appropriate controls.

The percentage of HPV-positive CIN2/3 cases was very high, as expected. The low proportion of HPV-negative samples among CIN2/3 cases is consistent with previous reports of HPV DNA-negative CIN2 and CIN3, even though an incorrect histological diagnosis was suspected [41-43]. Among controls, HPV positivity was about 20%, which is in agreement with the mean prevalence described in Brazilian populations (10.4%-24.5%) [44]. Frequency analysis of population characteristics and HPV infection were conducted in controls only, as almost all CIN2/3 cases were HPV-positive. This analysis confirmed the risk factors for HPV infection already described in the literature, including young age, low education level, smoking and a higher lifetime number of sexual partners. Brazilian Mixed, was the ethnic group that showed the highest prevalence of HPV infection, both in CIN2/3 cases and controls.

The analyses of HPV type distribution in our study population showed a slight increase in the prevalence of some types compared to the distribution that has been previously described in Brazil [24], but were in agreement with the reported prevalence for South America in a worldwide analysis of HPV type distribution [45].

To our knowledge, this is the first study on HLA-G methylation and its association with high-grade cervical lesions. We found a high mean percentage of methylation in both CIN2/3 cases and controls, without substantial differences. This is not in line with data reported for some other cancer types. In ovarian cancer, malignant cells were reported to show higher levels of methylation than normal control cells in some CpG sites, even though expression of the protein did not properly correlate with the methylation status [17]. In renal cancer cells, HLA-G expression via partial de-methylation of its promoter was counted among the strategies used by malignant cells to escape immune response [46]. Indeed HLA-G expression is widely documented in renal cancer cells, while no expression has been reported in normal renal cells [47-49]. Although HLA-G expression has been documented in several other cancer sites, i.e. cervical cancer [15,34-37], melanoma [49-51], breast [49,52-54], colorectal [55-57], gastric [57-60], esophageal [57,61,62], lung [57,63,64], and other cancers [49], the implications of HLA-G methylation on the expression of the protein have not been described.

It has been suggested that even a single CpG dinucleotide could represent a regulatory sequence highly predictive of the explored outcome [65]. Thus we also restricted our association analyses to two specific CpGs that might play a regulatory role, one located in the binding site of transcription factor Sp1, and one in the *enhancer* region. However, no significant differences were found in methylation between CIN2/3 cases and controls. Evidence of de-methylation events in CIN2/3 cases with respect to controls, which could suggest a re-expression of the HLA-G protein in the cells of cervical high-grade lesions, was not observed. If anything, we found a slight increase in the overall methylation percentage in CIN2/3 cases. This increase was more evident when the analysis was restricted to the CpG located in the *enhancer* region, specifically when a threshold was set for the methylation level. Although it seems paradoxical, this could be explained by the concomitant global DNA methylation induced by persistent HPV infection [66]. Recent studies have suggested that HPV can modulate DNA methylation patterns in order to control cell proliferation. The oncogenic HPV E7 protein can bind DNA methyltransferases, stimulating their activity [67]. Indeed, many genes have been shown to be hypermethylated in neoplastic cervical lesions [68]. We cannot exclude that the overlap of these hypermethylation events could overshadow low levels of de-methylation in the HLA-G promoter that may be present, or that may occur earlier in the carcinogenesis process. However the impact of low levels of de-methylation is unlikely to be functionally relevant.

Our findings of low HLA-G hypermethylation in CIN2/3 cases also suggested that alterations in methylation can be detected in the cervical samples of subjects with disease despite contamination of the sample by normal cells. This suggests that the results we obtained in our pilot analysis on HLA-G methylation are sufficiently suggestive of the absence of detectable de-methylation events in the HLA-G promoter, without requiring an extension of the analyses to the entire study population. Realistically, as has been previously reported, other mechanisms like histone modifications [69], polymorphisms [70] or miRNA [71] may modify HLA-G expression.

We compared population characteristics by HPV status and HLA-G promoter methylation, as some characteristics, including ethnicity, have been reported to affect either one or both of them [72]. If we had found an association, it would have been appropriate to evaluate our results in relation to the demographic and lifestyle characteristics of both cases and controls. However, while we could confirm known associations of some characteristics with HPV infection, we did not find any association with HLA-G methylation; we did not observe significant differences between CIN2/3 cases and controls, nor between HPV-positive and HPV-negative control women.

## Conclusions

This study did not support the hypothesis that spontaneous de-methylation events in the HLA-G promoter play a primary role in the development of precancerous cervical lesions through HLA-G re-expression and consequential promotion of viral and tumor escape from immunosurveillance. Nor was any association found between HLA-G methylation and various demographic and lifestyle factors.

## Abbreviations

CI: Confidence interval; CIN: Cervical intraepithelial neoplasia; HLA-G: Human leucocyte antigen-G; HPV: Human papillomavirus; LIGH: Laboratory of Immunogenetics and Hystocompatibility; OR: Odds ratio.

## Competing interests

The authors declare no competing interets.

## Authors' contributions

AGT, MGB, VF, LDM, HML, LR and FM participated in the design of the study. MBSX and RS extracted cervical DNA samples. AGT and VT set up the study database. NSC, CAM enrolled study women and provided cytological and histological diagnoses. VT, CG, MT, VF performed molecular analyses. AGT, VF, VT, LDM interpreted the results. LR, DZ performed statistical analyses. AGT drafted the manuscript. All authors read and approved the final manuscript.

## Pre-publication history

The pre-publication history for this paper can be accessed here:

http://www.biomedcentral.com/1471-2407/12/618/prepub
